# Estimates of possible severe bacterial infection in neonates in sub-Saharan Africa, south Asia, and Latin America for 2012: a systematic review and meta-analysis

**DOI:** 10.1016/S1473-3099(14)70804-7

**Published:** 2014-06-25

**Authors:** Anna C Seale, Hannah Blencowe, Alexander A Manu, Harish Nair, Rajiv Bahl, Shamim A Qazi, Anita K Zaidi, James A Berkley, Simon N Cousens, Joy E Lawn, for the pSBI Investigator Group

**Affiliations:** a Centre for Tropical Medicine, Nuffield Department of Medicine, University of Oxford, Oxford, UK; b KEMRI-Wellcome Trust Centre for Geographic Medicine and Research–Coast, Kilifi, Kenya; c Faculty of Epidemiology and Population Health UK, London School of Hygiene and Tropical Medicine, London, UK; d Centre for Maternal, Adolescent, Reproductive and Child Health, London School of Hygiene and Tropical Medicine, London, UK; e Centre for Population Health Sciences, University of Edinburgh, Edinburgh, UK; f Public Health Foundation of India, New Delhi, India; g Department of Maternal, Newborn, Child and Adolescent Health, World Health Organization, Geneva, Switzerland; h Department of Paediatrics and Child Health, Aga Khan University, Karachi, Pakistan; i Saving Newborn Lives/Save the Children, Washington, DC, USA

## Abstract

**Background:**

Bacterial infections are a leading cause of the 2·9 million annual neonatal deaths. Treatment is usually based on clinical diagnosis of possible severe bacterial infection (pSBI). To guide programme planning, we have undertaken the first estimates of neonatal pSBI, by sex and by region, for sub-Saharan Africa, south Asia, and Latin America.

**Methods:**

We included data for pSBI incidence in neonates of 32 weeks' gestation or more (or birthweight ≥1500 g) with livebirth denominator data, undertaking a systematic review and forming an investigator group to obtain unpublished data. We calculated pooled risk estimates for neonatal pSBI and case fatality risk, by sex and by region. We then applied these risk estimates to estimates of livebirths in sub-Saharan Africa, south Asia, and Latin America to estimate cases and associated deaths in 2012.

**Findings:**

We included data from 22 studies, for 259 944 neonates and 20 196 pSBI cases, with most of the data (18 of the 22 studies) coming from the investigator group. The pooled estimate of pSBI incidence risk was 7·6% (95% CI 6·1–9·2%) and the case-fatality risk associated with pSBI was 9·8% (7·4–12·2). We estimated that in 2012 there were 6·9 million cases (uncertainty range 5·5 million–8·3 million) of pSBI in neonates needing treatment: 3·5 million (2·8 million–4·2 million) in south Asia, 2·6 million (2·1 million–3·1 million) in sub-Saharan Africa, and 0·8 million (0·7 million–1·0 million) in Latin America. The risk of pSBI was greater in boys (risk ratio 1·12, 95% CI 1·06–1·18) than girls. We estimated that there were 0·68 million (0·46 million–0·92 million) neonatal deaths associated with pSBI in 2012.

**Interpretation:**

The need-to-treat population for pSBI in these three regions is high, with ten cases of pSBI diagnosed for each associated neonatal death. Deaths and disability can be reduced through improved prevention, detection, and case management.

**Funding:**

The Wellcome Trust and the Bill & Melinda Gates Foundation through grants to Child Health Epidemiology Reference Group (CHERG) and Save the Children's Saving Newborn Lives programme.

## Introduction

Severe bacterial infection in neonates, including sepsis, meningitis, and pneumonia, is an important contributor to the global burden of disease, accounting for about 3% of all disability-adjusted life years—a similar burden to that of HIV/AIDS.^1^, ^2^ Most of these disability-adjusted life years are attributable to deaths, because infections are a leading cause of the 2·9 million global neonatal deaths.^3^, ^4^ However, in addition to substantial immediate mortality, survivors of severe bacterial infection in the neonatal period (first 28 days of life) are at risk of long-term disability.^5^ Timely detection and appropriate case management could save hundreds of thousands of newborn lives.^6^ But, by contrast with HIV,^7^ childhood pneumonia,^8^ and malaria,^9^ no published estimates of the incidence of severe neonatal bacterial infection are available. This information is essential to guide health-programme priorities and policy in support of the post-2015 agenda to end preventable child deaths. It aligns closely with WHO's Every Newborn Action Plan,^10^ which sets a target of ten or fewer neonatal deaths per 1000 livebirths in every country by 2035.^11^

**Figure 1:**
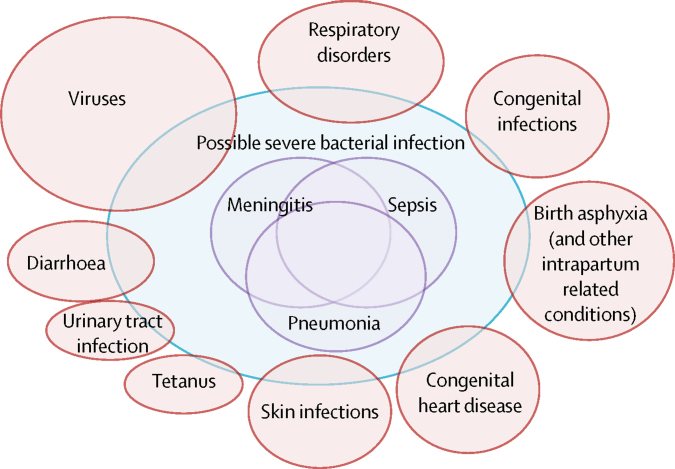
Possible severe bacterial infection (pSBI) and overlap with other clinical syndromes

The clinical diagnosis of severe neonatal bacterial infection is challenging, recognition of illness by a baby's primary caregiver can occur late, symptoms can be subtle and proceed rapidly, and signs can be non-specific and difficult to detect. In resource-poor settings, first-line care is rarely given by those with specialist paediatric training, and reliable microbiological investigations to support diagnoses are uncommon outside of research centres. Clinical algorithms have been developed to direct treatment of neonates identified with possible severe bacterial infection (pSBI) as in the WHO guidelines of the Integrated Management of Childhood Illness.^12^ This algorithm was initially informed by the first WHO Young Infants Study in the 1990s,^13^ which identified 14 clinical signs and symptoms predictive of severe bacterial disease. However, these guidelines excluded neonates in the first week of life, when the risk of infection and death are highest but clinical signs are less specific compared with older children. The subsequent WHO Young Infants Clinical Signs Study (YICSS),^12^ focused on clinical signs detected by primary care health workers for 3177 neonates in the first week of life attending health-care facilities in countries within sub-Saharan Africa, south Asia, and Latin America: Bangladesh, Bolivia,^14^ Ghana,^15^ India,^16^ Pakistan, and South Africa.^17^ The presence of any one of seven clinical signs and symptoms predicted severe bacterial illness (on the basis of an experienced paediatrician's assessment) with a sensitivity of 85% and specificity of 75%, in those seeking care.^12^ Due to the likely high mortality in neonates with severe bacterial infection who are not treated, clinical algorithms to diagnose pSBI prioritise sensitivity over specificity, so this diagnosis includes other disorders such as transient tachypnoea, hypoglycaemia, respiratory distress associated with preterm birth, birth asphyxia, and viral respiratory infections (figure 1).^18^

In both resource-poor and resource-rich settings, challenges exist in the diagnosis of severe neonatal bacterial infection. However, in rich countries, diagnosis is usually done by experienced paediatricians, and almost all cases of pSBI are investigated with a full sepsis screen (testing of blood, cerebrospinal fluid, and urine for signs of infection) and by haematological and biochemical investigations. Clinical algorithms are also used in resource-rich regions, but focus on the prediction of outcomes in very high-risk neonates; for example, the clinical risk index for babies, which assesses likelihood of hospital death in preterm neonates on the basis of gestational age, birthweight, maximum and minimum fraction of inspired oxygen, maximum base excess during the first 12 h, and presence of congenital malformations.^19^ Despite the additional expertise and investigations available in resource-rich settings, differentiation of bacterial illness from other disorders is difficult, and the improvement of diagnoses, for example through the use of novel markers of infection, is an important area of research.^20^

Estimation of the burden of pSBI is important to plan for health systems' response, in terms of staff, commodities, and, when required, inpatient care. There is variation in reported incidence of neonatal pSBI, which might be affected by differences in diagnostic criteria and case-finding strategies, as well as by true differences between populations.^21^ In studies from India,^22^, ^23^ Bangladesh,^24^, ^25^, ^26^ Pakistan,^27^ and Nepal,^28^, ^29^ pSBI incidence risks range from 50 cases to 217 cases per 1000 livebirths.

We aimed to estimate the burden of pSBI in neonates (excluding very preterm neonates, <32 weeks gestation or <1500 g birthweight) and associated mortality, in south Asia, sub-Saharan Africa, and Latin America in 2012. To do this we calculated pooled estimates of incidence risk and case-fatality risk for pSBI in neonates, by sex, by doing meta-analyses after a systematic review of published and unpublished data.

## Methods

### Search strategy and selection criteria

**Figure 2:**
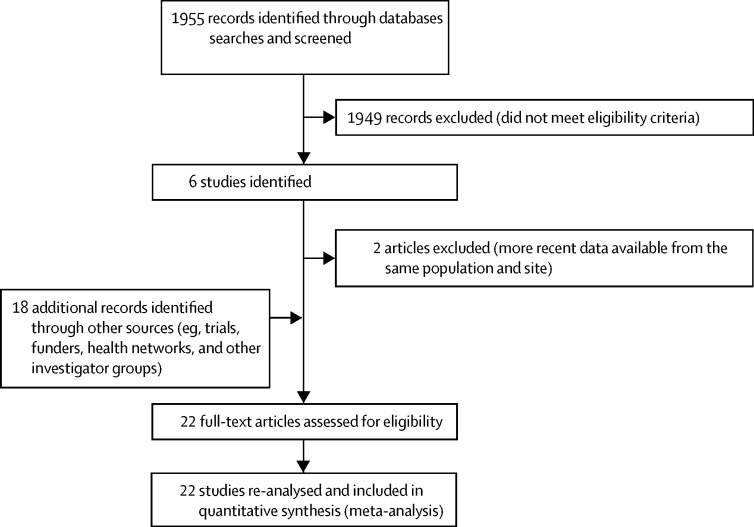
Data search and extraction^30^

We searched PubMed and WHO regional databases (AFRO, EMRO, Lilacs) to identify published data for incidence of pSBI (figure 2). We applied no language or date restrictions. Search terms included multiple variants of terms covering the areas “newborn/infant” and “infection” and we used Medical Subject Headings terms when available (see appendix for full details). We used snowball searching to identify further studies by screening the reference lists of retrieved studies. We did our last search on April 7, 2014.

We defined pSBI according to the YICSS criteria as the presence of any one of a history of difficulty feeding, history of convulsions, movement only when stimulated, respiratory rate of 60 or more breaths per min, severe chest indrawing, and a temperature of 37·5°C or higher or 35·5°C or lower.^12^ The details of the clinical signs and symptoms used for diagnosis in each included study are shown in the appendix.

Sub-Saharan Africa included Global Burden of Disease (GBD) sub-Saharan Africa regions central, east, west, and southern. South Asia included GBD regions south Asia and southeast Asia. Latin America included GBD regions central, Andean, southern, and tropical Latin America and the Caribbean.^31^

We searched for ongoing or recently completed unpublished clinical trials on ClinicalTrials.gov, and contacted Health and Demographic Surveillance Systems, and relevant research networks such as the Healthy Newborn Network and the Oxford tropical network. We also contacted funding agencies or their overseas centres (the Bill & Melinda Gates Foundation, Saving Newborn Lives, WHO, the Joint United Nations Programme on HIV/AIDS, the Wellcome Trust, and the Medical Research Council) to request data from studies meeting our inclusion criteria. We also contacted members of the investigator group contributing data to a study of the global burden of lower respiratory tract infections.^8^ We subsequently assembled the pSBI Investigator Group to include secondary analyses of data from published and as yet unpublished studies. Investigators were asked to reanalyse data using a common data template and analytic framework, when appropriate, and to join the pSBI Investigator Group.

To be included in our analysis, studies had to meet all inclusion criteria: they had to be recent (data obtained no earlier than 1998), a denominator for the population at risk had to be available, and diagnosis of pSBI had to be established on the basis of the presence of specified signs and symptoms. We excluded studies of preterm babies (<32 weeks gestation) and very low birthweight babies (<1500 g), and where possible excluded data for these neonates from secondary analyses, because death and disability in very preterm newborns is more a consequence of their prematurity than infection alone.

We did not exclude studies on the basis of their method of case ascertainment, but categorised them as active prospective case finding (community workers visit homes and assess neonates according to a schedule), active retrospective case finding (community workers visit homes and ask about signs and symptoms that occurred in the neonatal period), or passive case finding (recording attendance at a health centre). We categorised studies as such to allow assessment of the effect of different methods of case ascertainment on estimates of pSBI incidence risk, because studies with active follow-up would include neonates who might not have otherwise sought care, or might have died at home, by contrast with passive case finding, in which such cases would be missed. To further assess the effect of differing methods of case ascertainment, we categorised the frequency of visits in active, prospective case-finding studies into frequent visits (four or more visits during the neonatal period) or less-frequent visits (fewer than four visits during the neonatal period). We also differentiated between studies that included interventions and those that did not.

When possible, we re-analysed data to define pSBI cases according to the YICSS algorithm, but because this was not always possible, we categorised studies into those using the YICSS algorithm exactly, those using a modified YICSS algorithm, and those using substantially different criteria (see appendix for details).

Datasets analysed by the original study investigators were approved under the existing site's institutional review board. Further ethical review was not applicable for secondary data analysis of aggregated data from the pSBI investigator group.

### Statistical analysis

We calculated pooled estimates using random effects models (Der-Simoninan and Laird method).^32^ We used random effects models because they allow for heterogeneity across studies by use of a statistical parameter representing the variation between studies. We expected heterogeneity because of true differences in the pSBI incidence and associated mortality in study sites within the regions studied. These differences would be expected due to differing socioeconomic conditions, population comorbidities, and environments; some studies were done in predominantly urban areas, others were done in rural areas. Similarly, study design variations might have introduced heterogeneity. We assessed the extent to which results varied across studies using the *I*^2^ statistic, which estimates the proportion of variability between studies due to actual differences in results versus chance differences, with higher values suggesting more heterogeneity.^33^

We estimated incidence risk and case-fatality risk of pSBI overall, by region, and by sex. We further investigated the effect of sex by calculating a pooled estimate for the risk ratio for pSBI in both sexes. We used this pooled risk ratio to derive the incidence risk in both sexes from the overall incidence risk of pSBI, because not all the studies included data for sex.

We assessed the effect of case ascertainment method, frequency of home visits, diagnostic algorithm used, and whether or not there was an intervention, by calculating separate pooled estimates of pSBI incidence risk, looking at estimates within categories for each of these factors. We then did a sensitivity analysis, calculating a second pooled estimate of pSBI incidence risk, but including only those studies with active, prospective case ascertainment and using the YICSS algorithm (exact or modified) for diagnosis of pSBI.

We used a standard compartmental model,^2^ applying our pooled overall estimate of pSBI incidence risk to estimates of the number of livebirths (not including babies of <32 weeks gestation or <1500 g birthweight),^34^ by region and by sex in 2012.^35^ We applied pSBI incidence risks for both sexes (derived from the risk ratio as described above) to estimates of livebirths by region and by sex.^35^ We then applied the overall case-fatality risk to cases of pSBI, to provide an estimate of deaths after pSBI.

We derived a quantitative assessment of the uncertainty in the estimate of pSBI cases, and in consequent estimates of pSBI deaths, by taking 1000 random draws, assuming a normal distribution with a mean equal to the point estimate of the parameter and SD equal to the estimated SE of the parameter. This allowed for uncertainty in the number of cases to be captured in the estimation of the number of deaths. We calculated the 2·5th and 97·5th centiles of the resulting distributions as the uncertainty range.

We used Stata (version 12) for all statistical analyses.

### Role of the funding source

The study sponsors had no role in the study design, or in the collection, analysis, and interpretation of data, or in the writing of the report. ACS had full access to all the data in the study, and ACS, JEL, and HB had final responsibility for the decision to submit for publication.

## Results

**Figure 3:**
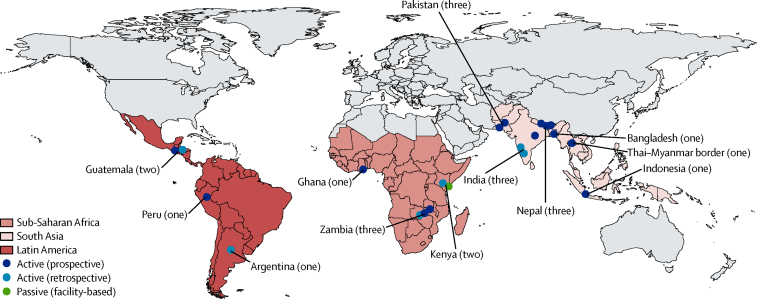
Geographical distribution of the 22 included studies The number of studies per country is shown in parentheses after country name.

Our systematic review identified 1955 abstracts (figure 2), from which we identified six eligible studies^24^, ^25^, ^26^, ^27^, ^28^, ^36^ meeting our inclusion criteria. We excluded two otherwise eligible studies^24^, ^25^ because more recent data were available from the same study site. We included data from an additional 18 studies identified from other sources (figure 3).^37^

We included 22 studies in the initial analysis (figure 2, table 1). The total study population included 259 944 neonates, with 20 196 cases of pSBI. 15 studies used active prospective case finding,^27^, ^37^, ^38^, ^40^, ^41^, ^42^, ^43^, ^44^, ^45^, ^46^, ^47^, ^48^ six studies used active retrospective case finding,^39^ and one study used passive case finding (unpublished). Eight studies used the YICSS algorithm exactly,^42^, ^45^, ^46^ eleven used modified YICSS criteria (eg, inclusion of omphalitis),^27^, ^37^, ^39^, ^40^, ^41^, ^43^, ^47^, ^48^ and three studies used criteria with important differences from the YICSS algorithm—these were the studies from the Thai–Myanmar border,^44^ India (Ballabgarh, Haryana state),^38^ and Peru (unpublished; see the appendix for details of study algorithms).

**Table 1:** Overview of the 22 included studies of possible severe bacterial infection

	**Country**	**Location**	**Rural or mixed (rural-urban)**	**Intervention**	**Neonatal mortality rate per 1000 livebirths**	**Year (median)**	**Period of data collection**	Algorithm*	**Case ascertainment**
Mitra et al (unpublished)	Bangladesh	Sylhet	Rural	Cord cleansing with chlorhexidine	28·3	2008	2007–09	YICSS	Visit on days 1, 3, 6, 9, and 15
Broor et al^38^	India	Ballabgarh, Haryana State	Rural	None	37	2003	2001–04	Danger sign or fast breathing, indrawing, nasal flaring, or grunting	Visit on day 7
MNHR^39^	India	Nagpur District	Mixed	None (health registry)	26·2	2012	2012	YICSS adapted	Visit on day 1 or 2 and on day 42
MNHR^39^	India	Belgaum district	Mixed	None (health registry)	26·2	2012	2012	YICSS adapted	Visit on day 1 or 2 and on day 42
Khanal et al^40^	Nepal	Morang District	Rural	Community care	33	2006	2005–07	YICSS adapted; included skin and umbilicus infection	Visit on days 1 and 60
Saville et al^37^	Nepal	Dhanusha	Rural	Community interventions	32·5	2009	2007–11	YICSS adapted to include umbilical and skin infection	Visit on days 1, 3, 14, and 28
Mullany et al^36^, ^41^	Nepal	Sarlahi District	Rural	Chlorhexidine skin and umbilicus cleansing	32·1	2004	2002–06	YICSS; any two criteria	Visit on days 1, 2, 3, 4, 6, 8, 10, 12, 14, 21, and 28
Zaidi et al^27^	Pakistan	Karachi	Mixed	Outpatient management of infection	45	2005	2003–08	YICSS adapted	Visit days 1, 3, 6, 15, and 30
Bhutta et al^42^	Pakistan	Matiari	Rural	None	51	2012	2011–12	YICSS	Visit on days 1, 3, 6, 9, and 15
Soofi et al (unpublished)	Pakistan	District Naushero Feroze	Rural	None	44	2011	2010–12	YICSS	Visit on days 1, 3, 6, 9, and 15
Simoes et al^43^	Indonesia	West Java District	Mixed	None	NA	1999	1998–2001	YICSS adapted (excluding convulsions, poor feeding)	Visit on days 7, 14, 21, and 28
Turner et al^44^	Thai–Myanmar border	Thai–Myanmar border	Rural	None	15·9	2009	2009–10	Fever or two signs of severe disease	Visit on days 7 and 28
Kirkwood et al^45^	Ghana	Brong-Ahafo region	Rural	Community care	32·3	2009	2008–09	YICSS	Three visits in first 7 days of life
Berkley et al (unpublished)	Kenya	Kilifi District	Rural	None	13·3	2010	2009–11	YICSS	Neonates admitted to district hospital
MNHR^39^	Kenya	Western Highlands	Rural	None (health registry)	15·5	2012	2012	YICSS adapted	Visit on day 1 or 2 and on day 42
Hamer et al (unpublished)	Zambia	Southern Province	Rural	Cord cleansing with chlorhexidine	14·9	2012	2011–13	YICSS adapted; including jaundice and umbilicus infection	Visits on day 1, 4, 10, and 28
MNHR^39^	Zambia	Chongwe and Kafue District	Mixed	None (health registry)	22·7	2012	2012	YICSS adapted	Visit on day 1 or 2 and on day 42
Gill et al^46^	Zambia	Lufwanyama District	Rural	Community care	30·4	2007	2006–08	YICSS	At routine contact for postpartum visits
MNHR^39^	Argentina	Corrientes and Santiago Districts	Rural	None (health registry)	8·2	2012	2012	YICSS adapted	Visit on days 1 or 2 and on day 42
MNHR^39^	Guatemala	Chimaltenango District	Mixed	None (health registry)	25·2	2012	2012	YICSS adapted	Visit on day 1 or 2 and on day 42
Bruce et al^47^, ^48^	Guatemala	Highlands	Rural	Reducing indoor air pollution	N/A	2008	2002–04	YICSS (except indrawing)	Visit days 7, 14, 21, and 28
Tinoco et al (unpublished)	Peru	Lima, Tumbes, Cuzco, Puerto Maldonado	Mixed	None	9	2010	2009–11	Influenza-like illness	Visit three times per week

MNHR=Maternal and Newborn Health Registry. NA=not applicable. YICSS=Young Infants Clinical Signs Study.

* See appendix for further information.

**Figure 4:**
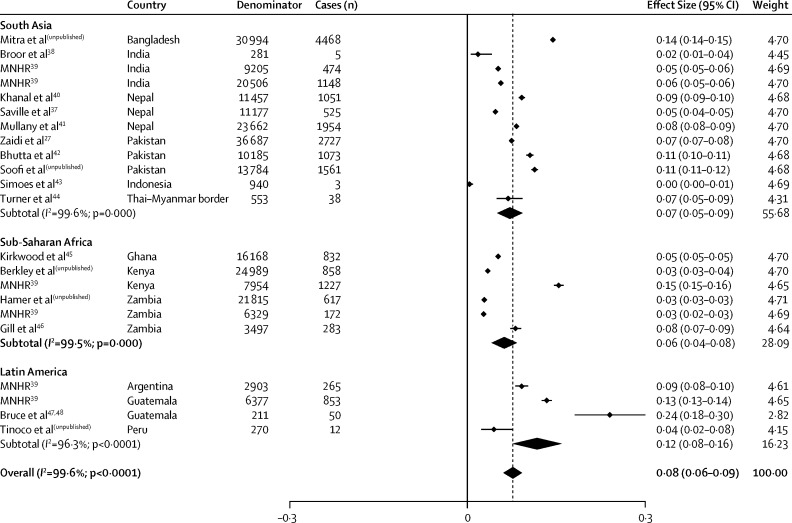
Meta-analysis for possible severe bacterial infection incidence, by region *As per random effects analysis.

The pooled estimate of pSBI incidence risk including all 22 studies was 7·6% (95% CI 6·1–9·2%; figure 4). We saw no evidence that estimated pSBI risk was higher in studies with prospective community follow-up than with retrospective community follow-up (see the appendix for this meta-analysis), and only one study used passive follow-up (facility-based). Among studies with prospective follow-up and four or more visits in the neonatal period, the estimated incidence risk of pSBI was 8·3% (5·5–11·1), and for those with fewer than four visits the incidence risk of pSBI was 6·3% (3·9–8·6). Studies including interventions did not have a lower incidence risk of pSBI (appendix). In terms of the algorithm used, the incidence risk reported in studies using the YICSS algorithm exactly was 8·2% (4·9–11·5) versus 8·1% (6·1–10·1) in those with a modified version and 4·3% (1·1–7·5) in those using substantially different criteria (appendix). When restricting the analysis to the 12 studies with active follow up and using the YICSS algorithm for diagnosis (sensitivity analysis), the pooled estimate for pSBI incidence risk was 8·4% (6·0–10·7; appendix).

Incidence risks by region are presented in table 2. Although the overall incidence risk of pSBI was within the 95% confidence limits for each region, we saw a higher point estimate of incidence in Latin America than south Asia and sub-Saharan Africa (table 2). We saw much heterogeneity between results of studies within regions (*I*^2^≥95%; figure 4).

**Table 2:** Summary pooled incidence risk estimates from meta-analyses, by region

		**South Asia**	**Sub-Saharan Africa**	**Latin America**	**Overall**
**Livebirths ≥32 weeks (n)**^35^					
Total		46 000 000	34 100 000	10 800 000	91 000 000
Boys		23 700 000	17 400 000	5 500 000	47 000 000
Girls		22 300 000	16 700 000	5 300 000	44 000 000
**Studies (n)**					
Study sites		12	6	4	22
Study population		169 431	80 752	9761	259 944
pSBI cases		15 027	3989	1180	20 196
pSBI incidence risk (95% CI)					
	Total	0·072 (0·050–0·093)	0·062 (0·041–0·083)	0·117 (0·076–0·159)	0·076 (0·061–0·092)
	Boys	0·085 (0.061–0·108)	0·069 (0·042–0·095)	0·149 (0·103–0·195)	0·089 (0·071–0·108)
	Girls	0·076 (0·055–0·097)	0·064 (0·038–0·090)	0·118 (0·076–0·160)	0·077 (0·062–0.096)
pSBI case fatality risk (95% CI)					
	Total	0·087 (0·056–0·118)	0·141 (0·072–0·210)	0·094 (0·063–0·125)	0·098 (0·074–0·122)
	Boys	0·093 (0·059–0·128)	0·137 (0·061–0·213)	0·111 (0·086–0·136)	0·103 (0·075–0·130)
	Girls	0·076 (0·047–0·104)	0·152 (0·061–0·242)	0·078 (0·033–0·122)	0·090 (0·065–0·114)

pBSI=possible severe bacterial infection.

**Figure 5:**
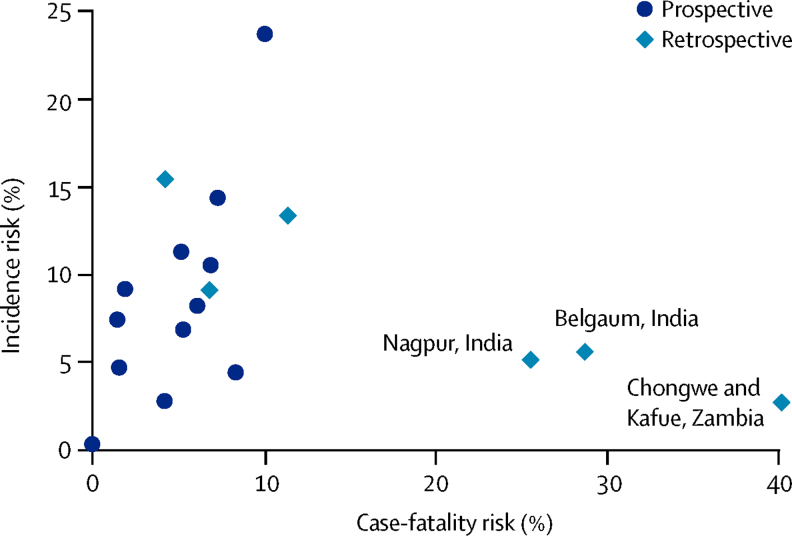
Scatter plot of risk of possible severe bacterial infection and case fatality risk, indicating method of case identification Outliers with a high case-fatality risk are labelled.

Overall, case-fatality risk associated with pSBI was 9·8% (95% CI 7·4–12·2; shown in table 2 by region; see appendix for the meta-analysis). Case-fatality risk estimates were higher in three studies,^39^ all with retrospective case-finding (figure 5), and again we saw much heterogeneity between studies, especially within south Asia and sub-Saharan Africa (*I*^2^≥97·8% for south Asia and sub-Saharan Africa; *I*^2^=43·7% for Latin America).

Boys had a higher risk of pSBI than did girls (data from 18 study sites): the risk ratio for pSBI in boys versus girls was 1·12 (1·06–1·18, see appendix for meta-analysis). All regions had increased risk in boys compared with girls, with this effect was most pronounced in Latin America (risk ratio 1·23, 95% CI 1·10–1·37) and little evidence of heterogeneity between the three study sites included.^39^, ^47^, ^48^ We saw no evidence for a difference in case-fatality risk between boys and girls with pSBI (1·06; 0·91–1·24; see appendix for meta-analysis).

Estimates of burden of disease were based on number of livebirths of 32 weeks gestation or more (or ≥1500 g birthweight) by country and sex in 2012 and by applying overall incidence risk of pSBI. We did not apply region-specific incidences for pSBI to livebirths by region because of the scarcity of data available for each region and the high heterogeneity between studies from the same region.

We estimated 6·9 million cases (uncertainty range 5·5–8·3) of pSBI in neonates among livebirths of 32 weeks gestation or more (or ≥1500 g birthweight) in 2012 (table 3).

**Table 3:** Estimates of cases of possible severe bacterial infection by region and sex

	**South Asia**	**Sub-Saharan Africa**	**Latin America**	Total*
**pSBI cases**				
Total in millions	3·5 (2·8–4·2)	2·6 (2·1–3·1)	0·8 (0·7–1·0)	6·9 (5·5–8·3)
Male cases in millions†	1·9 (1·5–2·4)	1·4 (1·1–1·8)	0·4 (0·4–0·6)	3·7 (3·0–4·7)
Female cases in millions†	1·6 (1·2–2·0)	1·2 (0·9–1·5)	0·4 (0·3–0·5)	3·2 (2·5–3·9)
**pSBI deaths**				
Total	340 000 (230 000–470 000)	250 000 (170 000–340 000)	00 000 (50 000–110 000)	680 000 (460 000–920 000)
Male cases‡	190 000 (130 000–260 000)	140 000 (90 000–190 000)	40 000 (30 000–50 000)	370 000 (280 000–460 000)
Female cases‡	160 000 (110 000–210 000)	120 000 (80 000–160 000)	40 000 (30 000–50 000)	310 000 (220 000–360 000)

Data are cases (uncertainty range). pBSI=possible severe bacterial infection.

* Totals for all regions are based on unrounded estimates of regional cases.

† Male and female pSBI cases calculated from pSBI incidence risk (table 2) adjusted for sex, assuming that male babies were at a 12% increased risk as per the risk ratio.

‡ Male and female deaths calculated from sex-specific pSBI cases and overall case-fatality risk.

## Discussion

Our estimate of 6·9 million cases (uncertainty range 5·5 million–8·3 million) of pSBI in south Asia, sub-Saharan Africa, and Latin America, suggests that both the size of the need-to-treat population and the burden of pSBI on health-care systems is substantial. To the best of our knowledge, these are the first estimates of neonatal pSBI cases, and due to the absence of comparable data in resource-rich regions, we have not been able to make global estimates; pSBI incidence risks and case-fatality risks probably differ. Because we know of no other first estimates of pSBI cases, we do not have other estimates for comparison. From these estimates we could, however, estimate associated deaths. Our mortality estimate of 680 000 neonatal deaths was based on only 22 studies, but is consistent with estimates of neonatal deaths due to infection from the Child Health Epidemiology Reference Group (CHERG), which estimated 640 000 worldwide in 2012,^11^ and Institute for Health Metrics Evaluation (IHME), which estimated 750 000 in 2010.^49^ The CHERG and IHME estimates are based on large datasets (more than a million deaths) drawn from vital registration and verbal autopsy studies, which report multicause mortality and are a more robust approach to cause-specific mortality estimates. That our estimates align closely with these lends support to the accuracy of our findings.

For the 6·9 million neonates with pSBI needing treatment in sub-Saharan Africa, south Asia, and South America, there are scarce data for how many were treated in health facilities, and for how many obtained antibiotics from commercial providers.^50^ A review of care seeking for neonatal illness in low-income and middle-income countries reported that a median of only 59% (range 10–100) of parents or guardians seek care for sick neonates, with most of the studies (17 [77%] of 22 studies) from Asia.^51^ In some societies, sick neonates, or indeed women who have recently given birth, are not given care,^52^ or care seeking is delayed, and a neonate with sepsis or meningitis might be dead within hours. Our analysis did not account for differing sociocultural behaviours, such as care seeking. However, the effect of such a difference was probably minimal because cases were identified for all but one study with active follow-up. We saw increased risk of infection and mortality in boys compared with girls, which is consistent with increased biological susceptibility in boys, as reported in resource-rich regions and in a study of acute lower respiratory tract infections.^8^, ^53^, ^54^ In south Asia, previously recorded higher female neonatal mortality has been attributed to reduced care seeking by families with female neonates who are sick,^55^, ^56^, ^57^ which might reverse the survival advantage for female neonates.

Our pooled data suggest that sub-Saharan Africa and south Asia have a similar incidence risk of pSBI (6·2% *vs* 7·2%), but that the case-fatality risk might be higher in sub-Saharan Africa (14·1% *vs* 8·7%). This finding could be due to chance, or due to variations in study design. However, there might also be real differences in access to and quality of care, and in risk factors such as preterm birth (higher in sub-Saharan Africa) or being small for gestational age (higher in south Asia),^2^, ^58^ or maternal infections such as HIV.^59^ More epidemiological data for pSBI and more consistency in diagnostic algorithms and case ascertainment in high-burden regions are needed to examine and understand any regional differences in pSBI incidence and case-fatality risk.

An important knowledge gap exists regarding the causes of severe neonatal bacterial infection in resource-poor settings. Pathogens such as *Streptococcus agalactiae* (group B streptococcus) and *Escherichia coli* are increasingly recognised as important in early onset sepsis in both resource-poor and resource-rich settings,^60^, ^61^ in addition to *Klebsiella pneumoniae* and *Staphylococcus aureus*.^62^ Important differences between settings include a probably higher incidence of coagulase negative staphylococcal infections in resource-rich regions in preterm neonates in hospital.^62^ Preterm neonates are more susceptible to infections,^63^, ^64^ and in resource-rich settings commonly have indwelling devices such as neonatal long lines, with which these infections are associated. Although extended admission to hospital increases exposure of neonates to hospital-acquired infection in resource-rich settings, hospital-acquired infections are also an increasing problem in resource-poor settings,^65^, ^66^ where anti-sepsis measures can be limited by resources and infrastructure. There is increasing evidence of antibiotic resistance,^65^ and more data for the pathogens and their susceptibilities are essential in understanding whether WHO treatment regimens are effective.^67^ Improvement of routine assessment and reporting of clinical case failures, as done for other diseases,^68^ could also inform assessment of the effectiveness of WHO guidelines. This is especially important in the context of ongoing research studies, which include testing strategies to simplify antibiotic treatment regimens and give treatments closer to a patient's home with administration by community health workers.^69^ Other strategies to reduce the burden of neonatal morbidity and mortality are diverse;^70^ those associated with reducing severe bacterial infection include improvement of supportive care through simple interventions such as kangaroo mother care.^71^, ^72^ However the best strategy is to prevent neonatal infection, which includes the development of maternal vaccinations,^73^ building on successful maternal vaccination programmes such as that for tetanus immunisation,^74^, ^75^ and improving hygiene. Clean delivery and chlorhexidine cord-cleansing at and after delivery have been shown to reduce neonatal mortality in randomised controlled trials in Pakistan, Nepal, and Bangladesh.^41^, ^76^, ^77^

Our study has important limitations, such as the scarcity of input data, with restricted data even within the three regions included (figure 3). We were unable to standardise for method of case finding; prospective case finding might identify cases with a lower case-fatality risk, compared with retrospective case finding (figure 5), probably due to reporting bias. Findings from a randomised controlled trial in Bangladesh, showed that neonatal pSBI diagnoses coincided with scheduled visits by community health-care workers.^24^ However, findings from this trial also showed that improved case finding through postnatal visits (together with antenatal care) reduced neonatal mortality by 39%.^24^ Visits were especially important at times of high risk (ie, the first 48 h of life).^78^ To what extent active prospective case finding leads to overdiagnosis of well neonates, or leads to earlier treatment, is unclear because both would reduce case-fatality risk.

We attempted to account for varied study diagnostic algorithms by reanalysis according to YICSS criteria when possible, but the specificity of diagnoses might be lower than reported in the YICSS study. In studies with prospective follow-up, the YICSS algorithm was used for screening in home-based surveillance by community health workers. However, in the YICSS study, the infants were mainly referred by their parents or guardians, and the studies were based in outpatient departments of large hospitals.^12^ If the specificity of the diagnostic algorithm was lower in the community than in YICSS, because the prevalence of pSBI in the community was low, the positive predictive value will be less than 50%, perhaps only 25%. Fast breathing, defined as a respiratory rate greater than 60 breaths per min in neonates, seems to have low specificity as a single sign.^79^ The presence of clinical signs might also depend on environmental determinants in the community; findings from a study in Nepal showed that 49% of all neonates had moderate hypothermia (32·0–36·0°C).^80^ Further research is needed to assess optimal diagnostic criteria for community screening of pSBI.

The high burden of pSBI in these three regions reinforces the urgent need for more investment, action, and innovation at all levels.^81^, ^82^ Improvement of prevention, minimisation of delays in recognition of illness, and instigation of rapid, appropriate management of pSBI is essential to reduce neonatal deaths and long-term disability. These actions are an important component of The Every Newborn Action Plan.^11^ Reducing mortality is rightly the main focus for action, even in the post-2015 era in view of the large number of preventable neonatal deaths. Treatment and innovation have been essential in reducing the HIV/AIDS burden, and now is the time to also invest in treatment and innovation for neonatal care.

**This online publication has been corrected. The corrected version first appeared at thelancet.com/infection on July 3, 2014**
