# Supplementary material for: Estimates of possible severe bacterial infection in neonates in sub-Saharan Africa, south Asia, and Latin America for 2012: a systematic review and meta-analysis
Source: Lancet Infect Dis. 2014 Jun 25;14(8):731–41. doi: 10.1016/S1473-3099(14)70804-7 (PMC4123782; doi:10.1016/S1473-3099(14)70804-7)

THE LANCET Infectious Diseases

Supplementary webappendix

This webappendix formed part of the original submission and has been peer reviewed. We post it as supplied by the authors.

Supplement to: Seale AC, Blencowe H, Manu AA, et al, for the pSBI Investigator Group. Estimates of possible severe bacterial infection in neonates in sub-Saharan Africa, south Asia, and Latin America for 2012: a systematic review and meta-analysis. *Lancet Infect Dis* 2014; published online June 26. http://dx.doi.org/10.1016/S1473-3099(14)70804-7.

This online publication has been corrected. The corrected version first appeared at thelancet.com/infection on July 3, 2014

Neonatal possible severe bacterial infection (pSBI) estimates in South Asia, sub-Saharan Africa and Latin America for 2012

WEB APPENDIX

Table of Contents

I Investigator group **2**

II Input data for estimation **4**

i) Search strategy and selection criteria **4**

ii) Signs and symptoms used for diagnoses **5**

iii) Pooled estimates **6**

a) Case fatality risk in pSBI cases, by region 6

b) Risk ratio of pSBI, comparing males and females, by region 7

c) Risk ratio of death in pSBI cases, comparing males and females, by region 8

d) Incidence risk of pSBI in males, by region 9

e) Incidence risk of pSBI in females, by region 10

III Sensitivity analyses **11**

f) Incidence risk of pSBI by method of case ascertainment 11

g) Incidence risk of pSBI categorised by number of neonatal visits, in studies with prospective follow up. 12

h) Incidence risk of pSBI categorised by algorithm used 13

i) Incidence risk of pSBI based on study type (intervention or non-intervention) 14

j) Sensitivity analysis: Incidence risk of pSBI using only studies with prospective community follow up and using YICSS algorithm (exact and modified). 15

I Investigator group

The pSBI Investigator Group: Dwi Agustian (PhD),[10] Fernando Althabe (MD),[11] Eduardo Azziz-Baumgartner (MD),[12] Rajiv Bahl (PhD),[6] Abdullah H Baqui (DrPH),[13] Daniel G. Bausch (MD),[14,19] Jose M. Belizan (PhD),[11] James A Berkley (MD),[1,2] Zulfiqar Bhutta (PhD),[15] Robert E Black (MD),[16] Shobha Broor (MD),[17] Hannah Blencowe (MRCPCH),[3] Nigel Bruce (PhD),[18] Pierre Buekens (PhD),[19] Harry Campbell (MD),[4] Waldemar A. Carlo (MD),[20] Elwyn Chomba (MD),[21] Anthony Costello (FRCPCH),[22] Simon N Cousens (PhD),[3] Richard J Derman (MD),[23] Mukesh Dherani (PhD),[18] Shams El-Arifeen (DrPH),[24] Cyril Engmann (MD),[25] Fabian Esamai (MMed),[26] Hammad Ganatra (MBBS),[7] Ana Garcés (MD),[27] Bradford D. Gessner (MD),[28] Christopher Gill (MD),[29] Robert L Goldenberg (MD),[30] Shivaprasad S Goudar (MD),[31] K. Michael Hambidge (MD),[32] Davidson H Hamer (MD),[33] Nellie I. Hansen (MPH),[34] Patricia L. Hibberd (MD),[35] Sudhir Khanal (MPH),[36] Betty Kirkwood (FMedSci),[3] Patrick Kosgei (HND),[2] Marion Koso-Thomas (MD),[37] Joy E Lawn (FRCPCH),[8,9] Edward A. Liechty (MD),[38] Alexander A Manu (PhD),[3] Elizabeth M McClure (PhD),[34] Dipak Mitra (PhD),[16] Neema Mturi (MRCPCH),[3] Luke C Mullany (PhD),[16] Harish Nair (PhD),[4,5] Charles R Newton (MD),[2, 39] Francois Nosten (PhD),[40] Shama Parveen (PhD),[41] Archana Patel (PhD),[42,43] Shamim A Qazi (MD),[6] Candice Romero (MD),[14] Naomi Saville (PhD),[22] Anna C Seale (MRCPCH),[1,2] Katherine Semrau (PhD),[33] Eric A.F. Simões (MD),[10,44] Sajid Soofi (FCPS),[15] Barbara J Stoll (MD),[45] Shiyam Sunder (MD),[15] Sana Syed (MBBS),[7] James M Tielsch (PhD),[16] Yeny O. Tinoco (PhD),[14] Claudia Turner (PhD),[40] Stefania Vergnano (MRCPCH),[46] Anita K Zaidi (MD).[7]

1. Centre for Tropical Medicine, Nuffield Department of Medicine, University of Oxford, Oxford, UK
2. KEMRI-Wellcome Trust Centre for Geographic Medicine and Research – Coast, Kilifi, Kenya
3. Faculty of Epidemiology and Population Health, London School of Hygiene and Tropical Medicine, Keppel St, London WC1E 7HT, UK
4. Centre for Population Health Sciences, University of Edinburgh, Edinburgh, EH8 9AG, UK
5. Public Health Foundation of India, New Delhi, India
6. World Health Organisation, Geneva
7. Department of Paediatrics and Child Health, Aga Khan University, Karachi, Pakistan.
8. Saving Newborn Lives / Save the Children, 2000 M Street NW, Suite 500,Washington, DC 20036, USA
9. Centre for Maternal, Adolescent, Reproductive & Child Health, London School of Hygiene and Tropical Medicine, Keppel St, London WC1E 7HT, UK
10. Department of Epidemiology & Biostatistics, Faculty of Medicine, Universitas Padjadjaran, Bandung, Indonesia
11. Departamento de Investigación en Salud de la Madre y el Niño, Instituto de Efectividad Clínica y Sanitaria Buenos Aires, Argentina
12. Influenza Division, Centers for Disease Control and Prevention, Atlanta, Georgia, U.S.A & International Centre for Diarrhoeal Disease Research, Bangladesh, Dhaka, Bangladesh
13. International Center for Maternal and Newborn Health, Johns Hopkins Bloomberg School of Public Health
14. Virology and Emerging Infections Department, U.S. Naval Medical Research Unit No. 6, Lima, Peru.
15. Department of Paediatrics and Child Health, The Aga Khan University, Pakistan
16. Department of International Health, Johns Hopkins Bloomberg School of Public Health, Baltimore, Maryland, United States of America
17. Department of Microbiology, All India Institute of Medical Sciences, New Delhi. PIN- 110029
18. Department of Public Health & Policy, University of Liverpool
19. Tulane School of Public Health and Tropical Medicine, New Orleans, LA
20. Department of Pediatrics, University of Alabama at Birmingham, Birmingham, Alabama.
21. University Teaching Hospital, Lusaka, Zambia.
22. UCL Institute for Global Health, London, UK
23. Department of Obstetrics and Gynecology, Christiana Care, Newark, Denver
24. International Centre for Diarrhoeal Disease Research, Bangladesh
25. Bill and Melinda Gates Foundation, 440 5th Ave N., Seattle, WA 98109, USA
26. Moi University, Eldoret, Kenya
27. Francisco Marroquin University, Guatemala City, Guatemala
28. Agence de Medicine Preventive, Paris, France
29. Department of International Health, Boston University School of Public Health
30. Obstetrics and Gynaecology, Columbia University, New York
31. Women's and Children's Health Research Unit, KLE University's J N Medical College, Belgaum, India
32. University of Colorado School of Medicine, Aurora, Colorado 80045
33. Center for Global Health and Development, Boston University, Boston, USA.
34. Social, Statistical and Environmental Sciences, RTI International, Research Triangle Park, NC.
35. Division of Global Health, Department of Pediatrics, Massachusetts General Hospital, Boston, Massachusetts 02114
36. MINI program, JSI R&T Nepal, Lalitpur Nepal
37. Eunice Kennedy Shriver National Institute of Child Health and Human Development Bethesda, Maryland
38. Indiana University School of Medicine, Indianapolis, Indiana, USA
39. Department of Psychiatry, University of Oxford, Oxford, UK
40. Shoklo Malaria Research Unit, Mahidol-Oxford Tropical Medicine Research Unit, Faculty of Tropical Medicine, Mahidol University, Mae Sot, Thailand
41. Centre for Interdisciplinary Research in Basic Sciences, Jamia Millia Islamia, New Delhi- 110025.
42. Indira Gandhi Government Medical College, Nagpur, India
43. Lata Medical Research Foundation, Nagpur, India.
44. Department of Pediatrics, University of Colorado and Center for Global Health, Department of Epidemiology, Colorado School of Public Health, Aurora Colorado, USA
45. Emory and Children's Healthcare of Atlanta, Emory University School of Medicine, Atlanta, Georgia, USA
46. St George's Hospital, Blackshaw Rd, Tooting, London, UK

II Input data for estimation

i) Search strategy and selection criteria

Neonatal terms ("infant, newborn"[MeSH Terms] OR ("infant"[All Fields] AND "newborn"[All Fields]) OR "newborn infant"[All Fields] OR "neonate"[All Fields]) OR ("infant, newborn"[MeSH Terms] OR ("infant"[All Fields] AND "newborn"[All Fields]) OR "newborn infant"[All Fields] OR "newborn"[All Fields])

AND

Infection terms; "Tetanus"[Mesh] OR "tetanus"[All Fields]; OR “Meningitis” [Mesh] OR "meningitis"[All Fields]; OR "Sepsis"[Mesh] OR "sepsis"[All Fields]; OR "Pneumonia"[Mesh] OR "pneumonia"[All Fields] OR "infection"[Mesh] OR "infection"[All Fields].

AND

Incidence terms; (rate [All Fields] OR ("epidemiology"[Subheading] OR "epidemiology"[All Fields] OR "incidence"[All Fields] OR "incidence"[MeSH Terms]))

AND

Community terms; population-based[All Fields] OR ("residence characteristics"[MeSH Terms] OR ("residence"[All Fields] AND "characteristics"[All Fields]) OR "residence characteristics"[All Fields] OR "community"[All Fields])

ii) Signs and symptoms used for diagnoses

Study	Country	Feeding difficulty	Lethargy/dec consc level	Tachypnoea	Fever	Hypothermia	Severe indrawing	Convulsion	Difficulty breathing	Severe diarrhoea /vomiting	Skin/ umbilical infection	Weak cry	Grunting	capillary refill > 2 secs	Nasal flaring	cough	sore throat	rhinorrhoea	Glucose imbalance	Abdo distension	Bilious aspirates	Blood in stool	Jaundice
**Study no.**	YICSS	√	√	√	√	√	√	√															
**1**	Bangladesh	√	√	√	√	√	√	√															
**2**	India	√	√	√			√	√		√			√		√								
**3**	India	√			√	√		√	√		√												
**4**	India	√			√	√		√	√		√												
**5**	Nepal	√	√	√	√	√					√	√											
**6**	Nepal	√	√	√	√	√	√				√	√	√										
**7**	Nepal*	√	√	√	√	√	√	√															
**8**	Pakistan	√	√		√	√	√	√					√	√									
**9**	Pakistan	√	√	√	√	√	√	√															
**10**	Pakistan	√	√	√	√	√	√	√															
**11**	Indonesia		√	√	√	√	√																
**12**	Thai-Myanmar			√	√									√					√	√	√	√	
**13**	Ghana	√	√	√	√	√	√	√															
**14**	Kenya	√	√	√	√	√	√	√															
**15**	Kenya	√			√	√		√	√		√												
**16**	Zambia	√	√		√	√		√	√		√												√
**17**	Zambia	√			√	√		√	√		√												
**18**	Zambia	√	√	√	√	√	√	√															
**19**	Argentina	√			√	√		√	√		√												
**20**	Guatemala	√			√	√		√	√		√												
**21**	Guatemala	√	√	√	√	√		√															
**22**	Peru				√											√	√	√					

***any two criteria**

iii) Pooled estimates

a) Case fatality risk in pSBI cases, by region

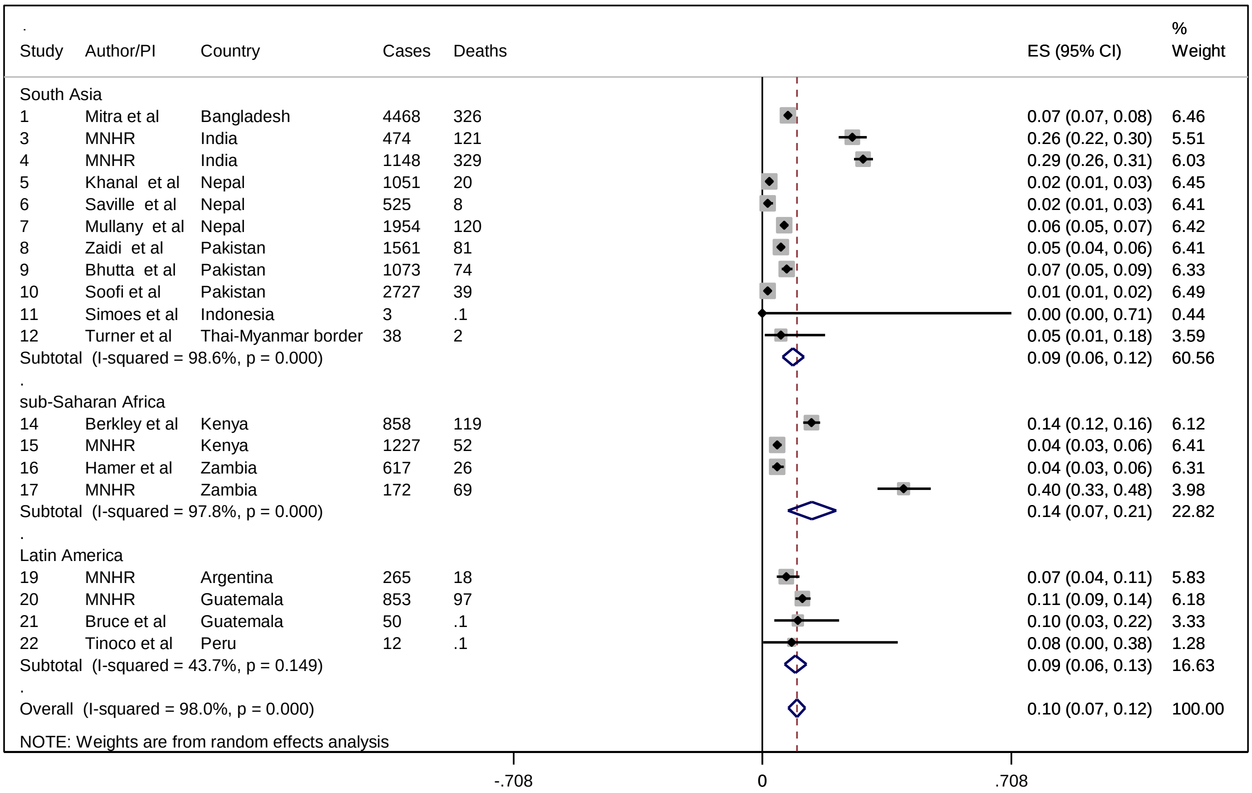

b) Risk ratio of pSBI, comparing males and females, by region

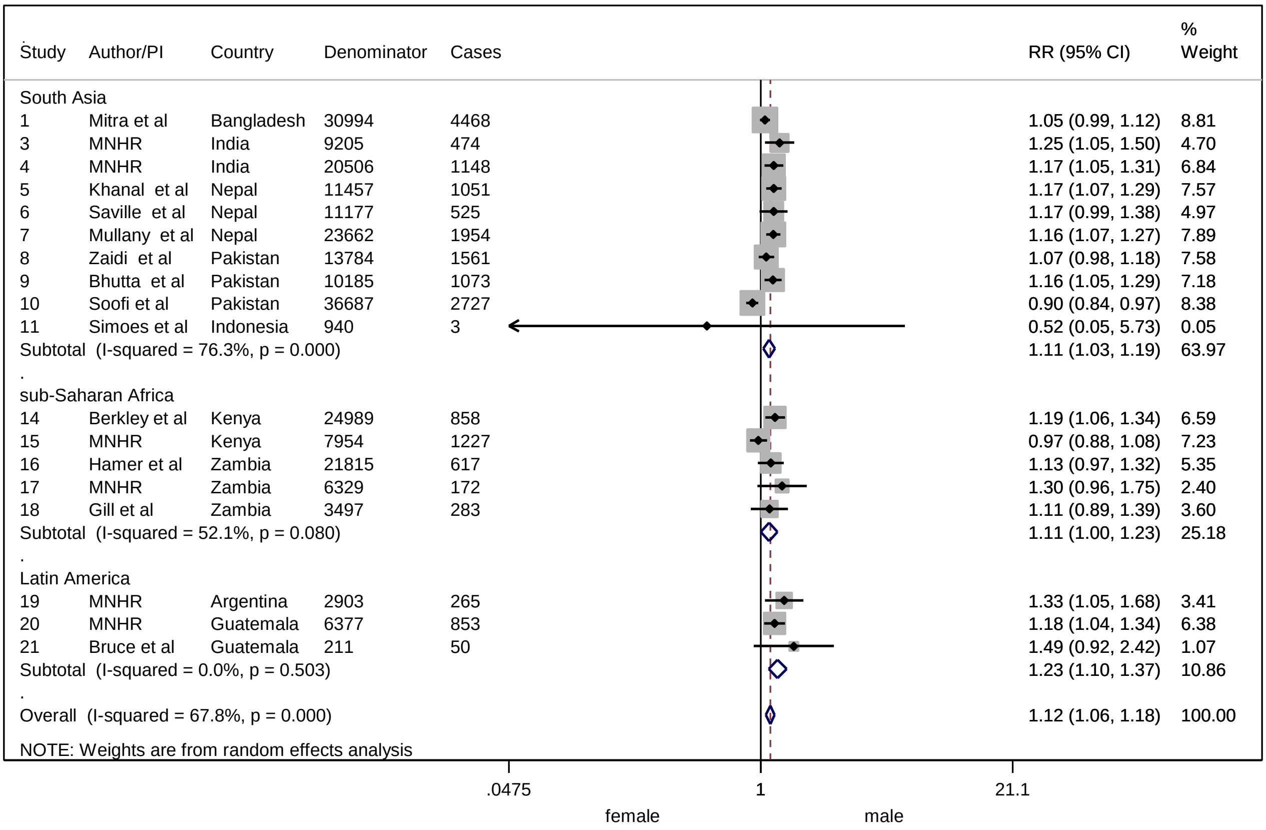

c) Risk ratio of death in pSBI cases, comparing males and females, by region

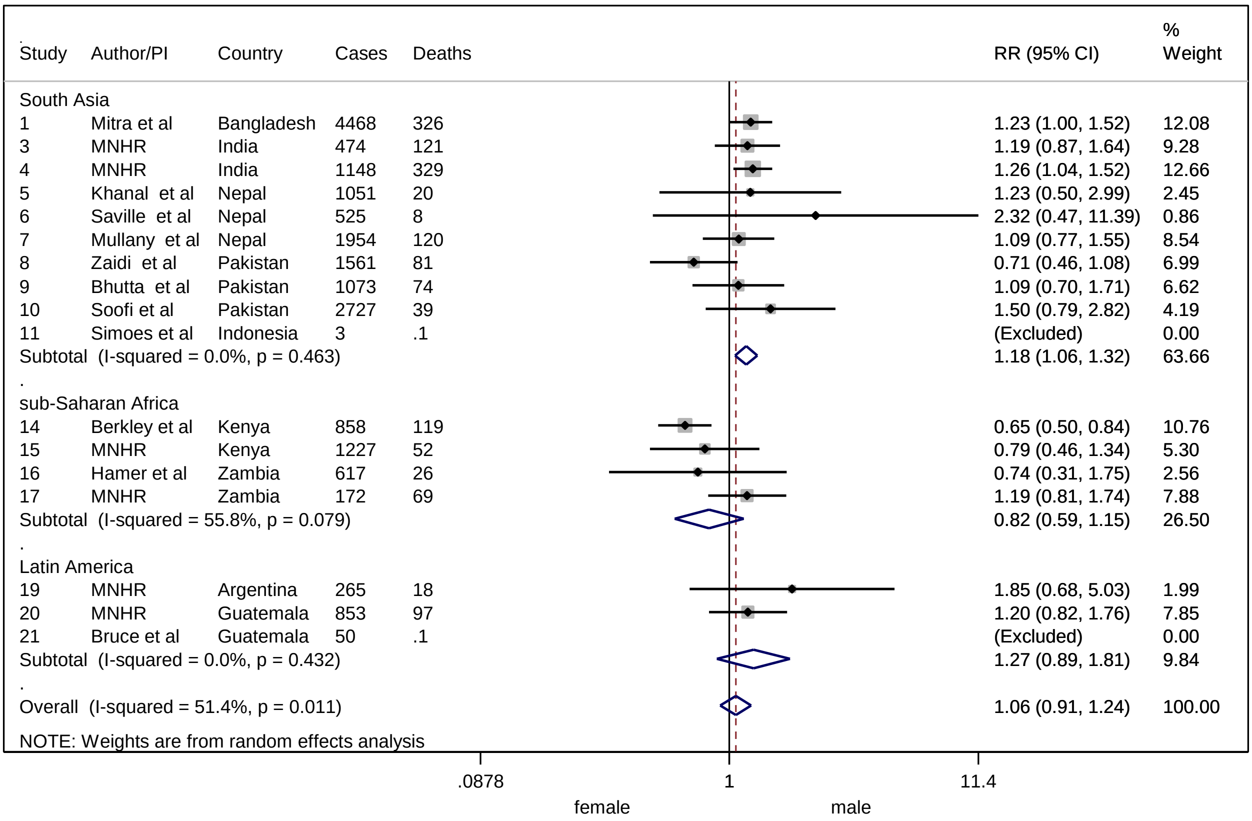

d) Incidence risk of pSBI in males, by region

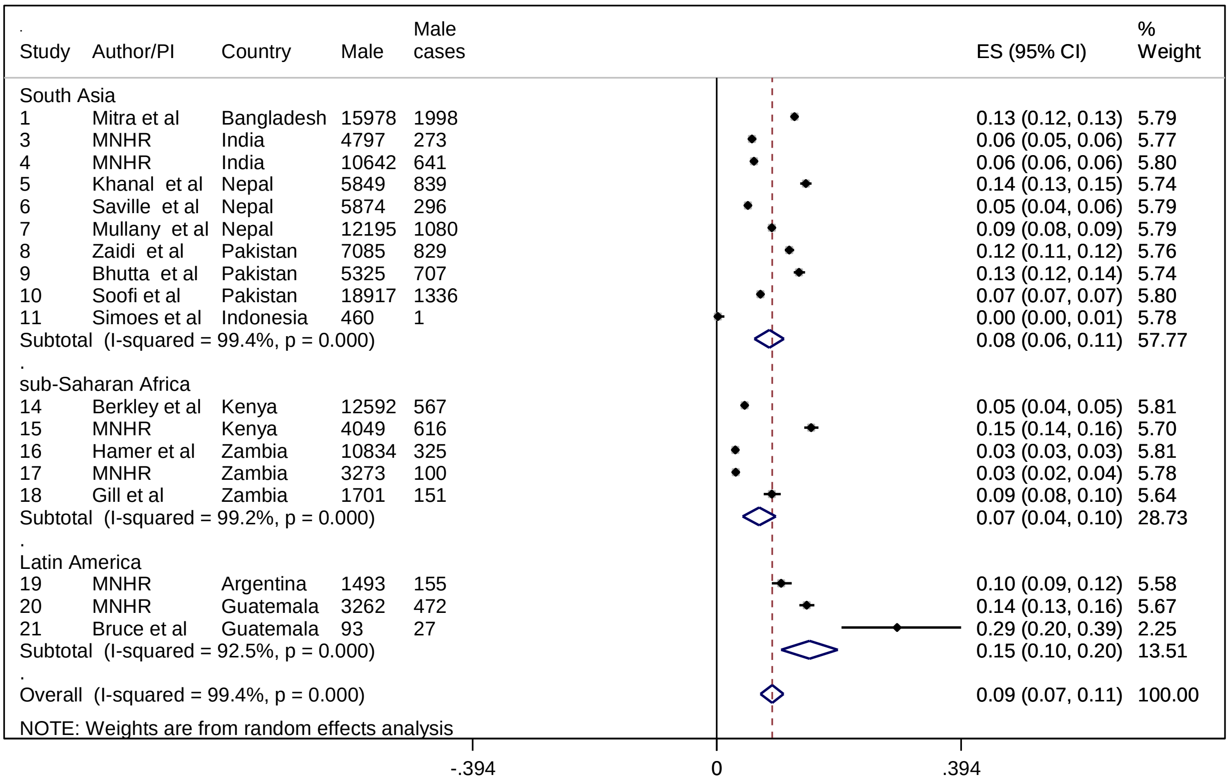

e) Incidence risk of pSBI in females, by region

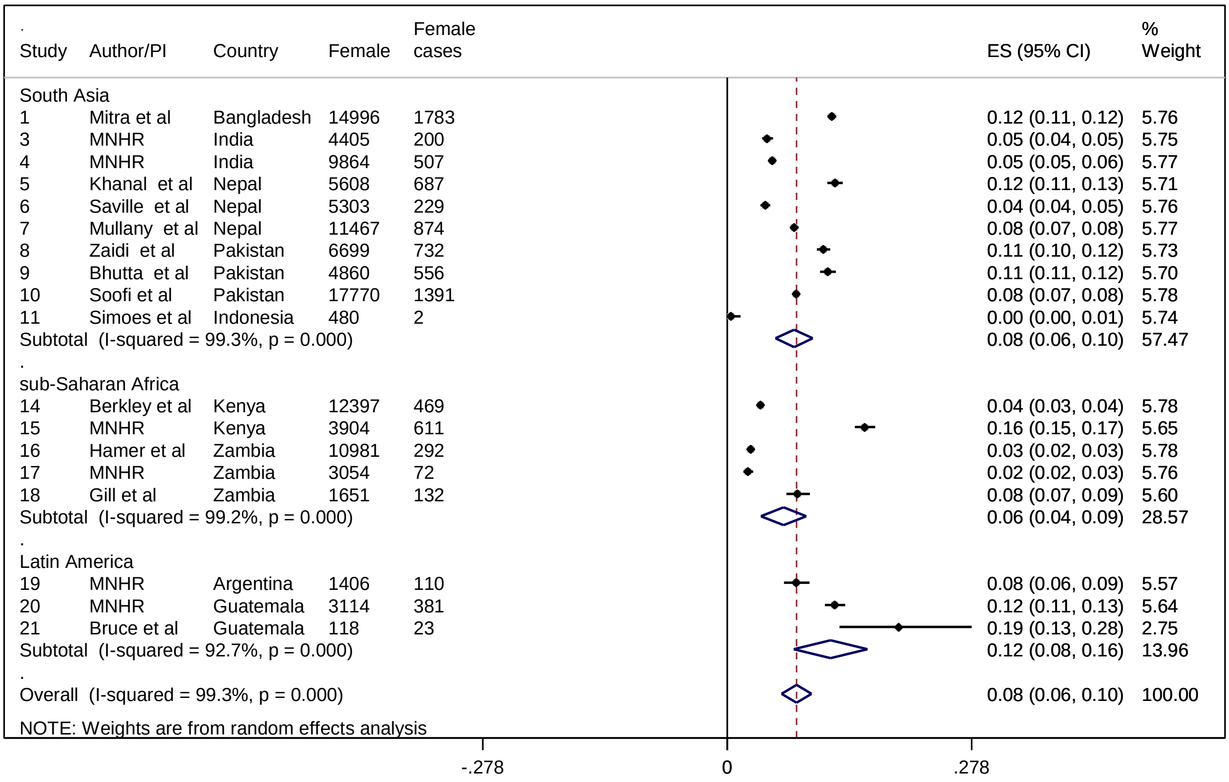

III Sensitivity analyses

f) Incidence risk of pSBI by method of case ascertainment

Study	Author/PI	Country	Denominator	Cases	ES (95% CI)	% Weight
Community, prospective						
1	Mitra et al	Bangladesh	30994	4468	0.14 (0.14, 0.15)	4.70
2	Broor et al	India	281	5	0.02 (0.01, 0.04)	4.45
5	Khanal et al	Nepal	11457	1051	0.09 (0.09, 0.10)	4.68
6	Saville et al	Nepal	11177	525	0.05 (0.04, 0.05)	4.70
7	Mullany et al	Nepal	23662	1954	0.08 (0.08, 0.09)	4.70
8	Zaidi et al	Pakistan	13784	1561	0.11 (0.11, 0.12)	4.68
9	Bhutta et al	Pakistan	10185	1073	0.11 (0.10, 0.11)	4.68
10	Soofi et al	Pakistan	36687	2727	0.07 (0.07, 0.08)	4.70
11	Simoes et al	Indonesia	940	3	0.00 (0.00, 0.01)	4.69
12	Turner et al	Thai-Myanmar border	553	38	0.07 (0.05, 0.09)	4.31
13	Kirkwood et al	Ghana	16168	832	0.05 (0.05, 0.05)	4.70
16	Hamer et al	Zambia	21815	617	0.03 (0.03, 0.03)	4.71
18	Gill et al	Zambia	3497	283	0.08 (0.07, 0.09)	4.64
21	Bruce et al	Guatemala	211	50	0.24 (0.18, 0.30)	2.82
22	Tinoco et al	Peru	270	12	0.04 (0.02, 0.08)	4.15
Subtotal (I-squared = 99.7%, p = 0.000)					0.08 (0.06, 0.10)	67.30
Community, retrospective						
3	MNHR	India	9205	474	0.05 (0.05, 0.06)	4.69
4	MNHR	India	20506	1148	0.06 (0.05, 0.06)	4.70
15	MNHR	Kenya	7954	1227	0.15 (0.15, 0.16)	4.65
17	MNHR	Zambia	6329	172	0.03 (0.02, 0.03)	4.69
19	MNHR	Argentina	2903	265	0.09 (0.08, 0.10)	4.61
20	MNHR	Guatemala	6377	853	0.13 (0.13, 0.14)	4.65
Subtotal (I-squared = 99.6%, p = 0.000)					0.09 (0.05, 0.12)	28.00
Facility based						
14	Berkley et al	Kenya	24989	858	0.03 (0.03, 0.04)	4.70
Subtotal (I-squared = .%, p = .)					0.03 (0.03, 0.04)	4.70
Overall (I-squared = 99.6%, p = 0.000)					0.08 (0.06, 0.09)	100.00

NOTE: Weights are from random effects analysis

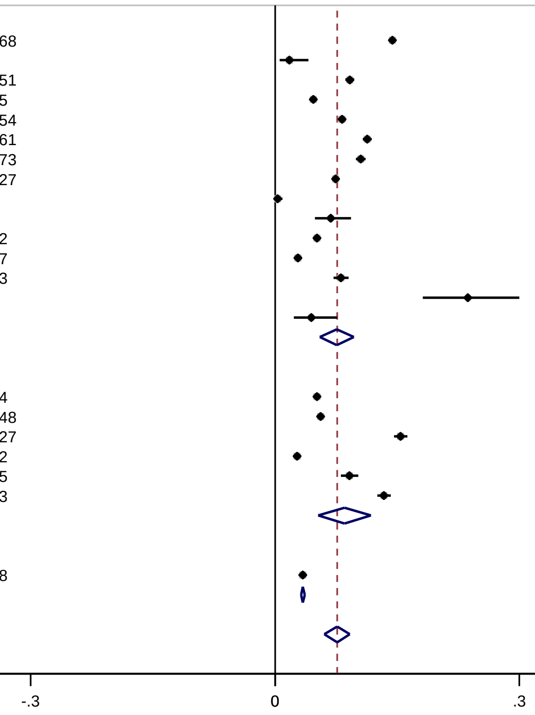

g) Incidence risk of pSBI categorised by number of neonatal visits, in studies with prospective follow up.

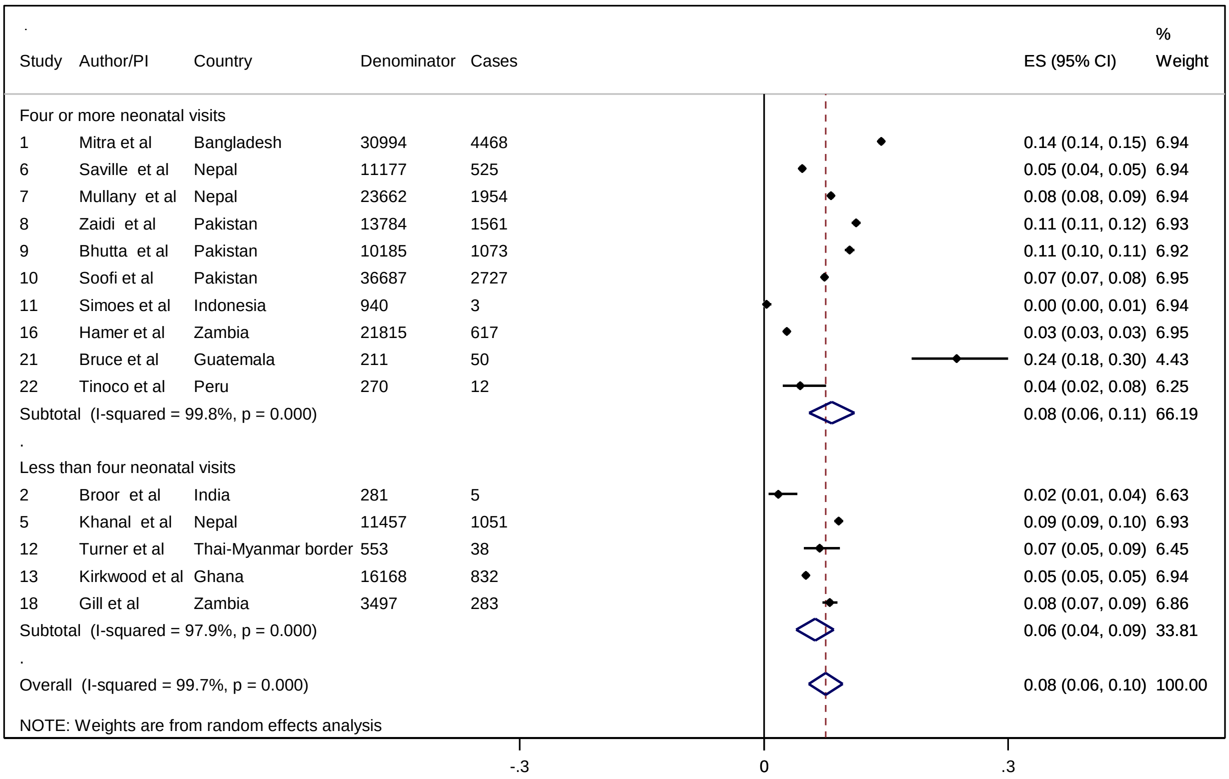

h) Incidence risk of pSBI categorised by algorithm used

Study	Author/PI	Country	Denominator	Cases	ES (95% CI)	% Weight
YICSS						
1	Mitra et al	Bangladesh	30994	4468	0.14 (0.14, 0.15)	4.70
9	Bhutta et al	Pakistan	10185	1073	0.11 (0.10, 0.11)	4.68
10	Soofi et al	Pakistan	36687	2727	0.07 (0.07, 0.08)	4.70
13	Kirkwood et al	Ghana	16168	832	0.05 (0.05, 0.05)	4.70
14	Berkley et al	Kenya	24989	858	0.03 (0.03, 0.04)	4.70
18	Gill et al	Zambia	3497	283	0.08 (0.07, 0.09)	4.64
Subtotal (I-squared = 99.8%, p = 0.000)					0.08 (0.05, 0.11)	28.12
.						
Not YICSS						
2	Broor et al	India	281	5	0.02 (0.01, 0.04)	4.45
12	Turner et al	Thai-Myanmar border	553	38	0.07 (0.05, 0.09)	4.31
22	Tinoco et al	Peru	270	12	0.04 (0.02, 0.08)	4.15
Subtotal (I-squared = 84.3%, p = 0.002)					0.04 (0.01, 0.07)	12.91
.						
YICSS-adapted						
3	MNHR	India	9205	474	0.05 (0.05, 0.06)	4.69
4	MNHR	India	20506	1148	0.06 (0.05, 0.06)	4.70
5	Khanal et al	Nepal	11457	1051	0.09 (0.09, 0.10)	4.68
6	Saville et al	Nepal	11177	525	0.05 (0.04, 0.05)	4.70
7	Mullany et al	Nepal	23662	1954	0.08 (0.08, 0.09)	4.70
8	Zaidi et al	Pakistan	13784	1561	0.11 (0.11, 0.12)	4.68
11	Simoes et al	Indonesia	940	3	0.00 (0.00, 0.01)	4.69
15	MNHR	Kenya	7954	1227	0.15 (0.15, 0.16)	4.65
16	Hamer et al	Zambia	21815	617	0.03 (0.03, 0.03)	4.71
17	MNHR	Zambia	6329	172	0.03 (0.02, 0.03)	4.69
19	MNHR	Argentina	2903	265	0.09 (0.08, 0.10)	4.61
20	MNHR	Guatemala	6377	853	0.13 (0.13, 0.14)	4.65
21	Bruce et al	Guatemala	211	50	0.24 (0.18, 0.30)	2.82
Subtotal (I-squared = 99.6%, p = 0.000)					0.08 (0.06, 0.10)	58.97
.						
Overall (I-squared = 99.6%, p = 0.000)					0.08 (0.06, 0.09)	100.00

NOTE: Weights are from random effects analysis

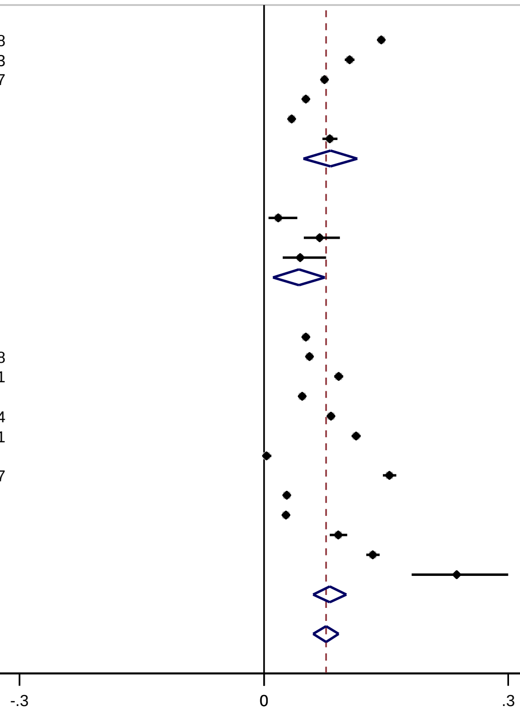

i) Incidence risk of pSBI based on study type (intervention or non-intervention)

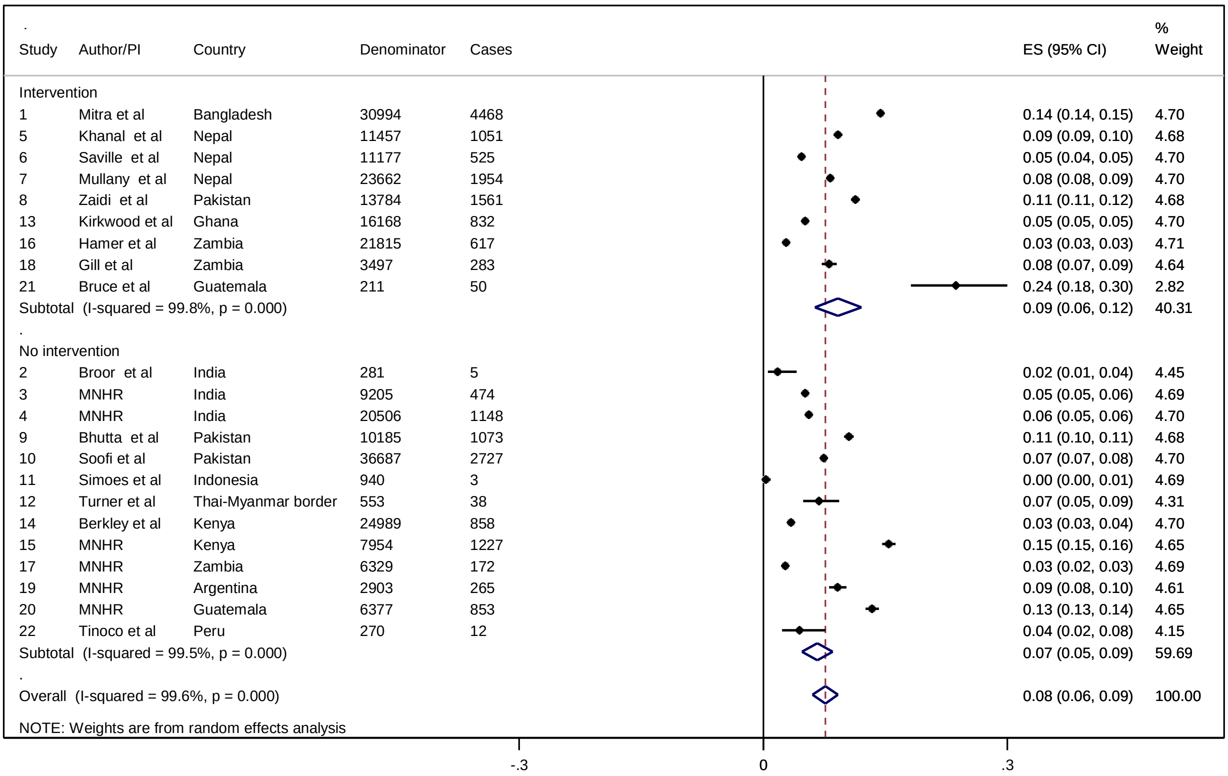

j) Sensitivity analysis: Incidence risk of pSBI using only studies with prospective community follow up and using YICSS algorithm (exact and modified).

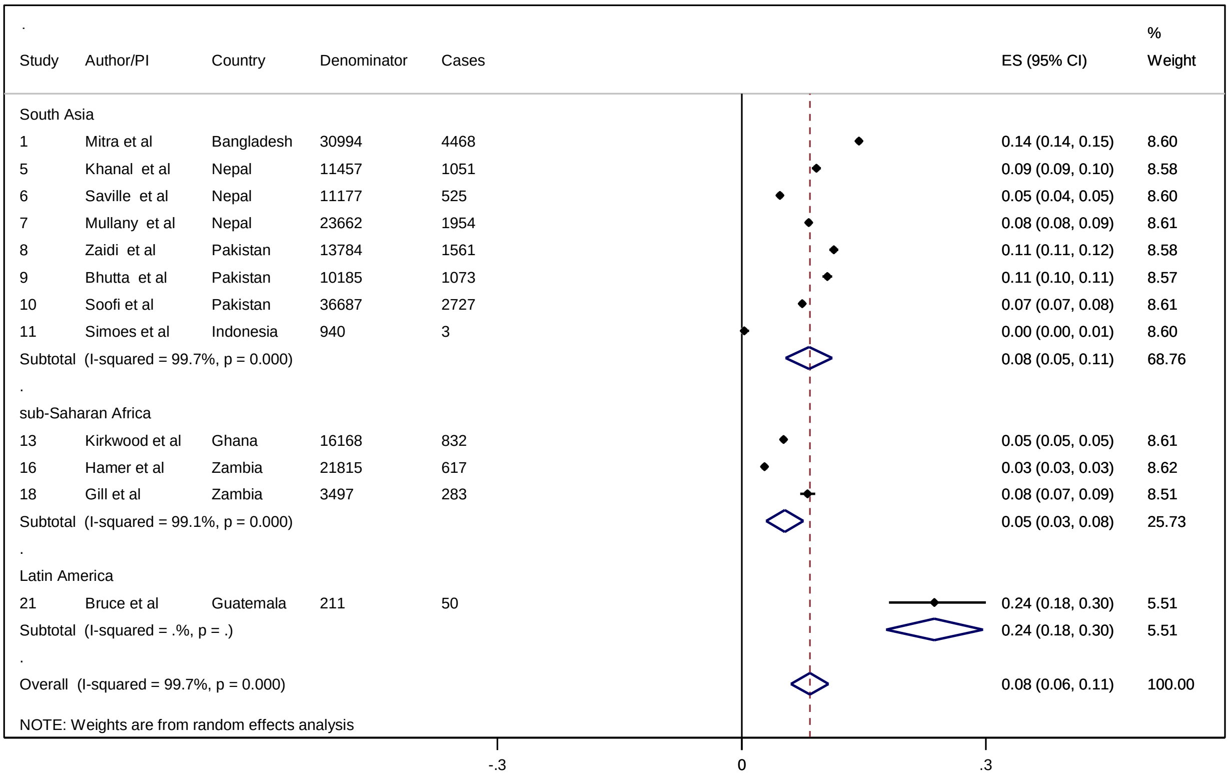

Supplement: Supplementary appendix [file mmc1.pdf]
